# Supplementary material for: An open-source framework for large-scale, flexible evaluation of biomedical text mining systems
Source: J Biomed Discov Collab. 2008 Jan 29;3:1. doi: 10.1186/1747-5333-3-1 (PMC2276192; doi:10.1186/1747-5333-3-1)
Supplement: Additional file 1 — Evaluation platform source code. The additional file contains the software evaluation framework discussed in this paper. The most current release can be downloaded from SourceForge.net [1]. See the accompanying README file for instructions on installation and use. Also included in the distribution is a collection of UIMA wrappers for some commonly used BioNLP tools and annotated corpora. [file 1747-5333-3-1-S1.zip › bionlp_uima_repository-1.0.1/docs/api/edu/uchsc/ccp/util/nlp/annotation/printer/DefaultPrintOutput.html]

DefaultPrintOutput (Center for Computational Pharmacology Util/UIMA API)


|  |  |  |
| --- | --- | --- |
| **Overview** \| **Package** \| **Class** \| **Tree** \| **Deprecated** \| **Index** \| **Help** | |  |
| **PREV CLASS**   **NEXT CLASS** | **FRAMES**    **NO FRAMES**     **All Classes** |  |
| SUMMARY: NESTED \| FIELD \| CONSTR \| METHOD | DETAIL: FIELD \| CONSTR \| METHOD |  |


---


## edu.uchsc.ccp.util.nlp.annotation.printer Class DefaultPrintOutput

```
java.lang.Object
  edu.uchsc.ccp.util.nlp.annotation.printer.DefaultPrintOutput
```

**All Implemented Interfaces:**: AnnotationPrinterUtil

---

``` public class DefaultPrintOutput extends Object implements AnnotationPrinterUtil ```

---

**Constructor Summary**

`DefaultPrintOutput()`


| **Method Summary** | |
| --- | --- |
| `void` | `printDocument(GenericDocument gd)` |
| `void` | `printDocument(GenericDocument gd, PrintStream ps)` |
| `void` | `printDocumentCollection(List<GenericDocument> documents)` |
| `void` | `printDocumentCollection(List<GenericDocument> documents, PrintStream ps)` |
| `void` | `setUseOneLineFormatStatus(boolean useOneLineFormat)` |

**Methods inherited from class java.lang.Object**

`clone, equals, finalize, getClass, hashCode, notify, notifyAll, toString, wait, wait, wait`

**Constructor Detail**

### DefaultPrintOutput

```
public DefaultPrintOutput()
```


**Method Detail**

### printDocumentCollection

```
public void printDocumentCollection(List<GenericDocument> documents)
```

:   **Specified by:**: `printDocumentCollection` in interface `AnnotationPrinterUtil`

---


### printDocumentCollection

```
public void printDocumentCollection(List<GenericDocument> documents,
                                    PrintStream ps)
```

:   **Specified by:**: `printDocumentCollection` in interface `AnnotationPrinterUtil`

---


### printDocument

```
public void printDocument(GenericDocument gd)
```

:   **Specified by:**: `printDocument` in interface `AnnotationPrinterUtil`

---


### setUseOneLineFormatStatus

```
public void setUseOneLineFormatStatus(boolean useOneLineFormat)
```

---


### printDocument

```
public void printDocument(GenericDocument gd,
                          PrintStream ps)
```

:   **Specified by:**: `printDocument` in interface `AnnotationPrinterUtil`


---


|  |  |  |
| --- | --- | --- |
| **Overview** \| **Package** \| **Class** \| **Tree** \| **Deprecated** \| **Index** \| **Help** | |  |
| **PREV CLASS**   **NEXT CLASS** | **FRAMES**    **NO FRAMES**     **All Classes** |  |
| SUMMARY: NESTED \| FIELD \| CONSTR \| METHOD | DETAIL: FIELD \| CONSTR \| METHOD |  |


---
